# Human Aquaporin 4 Gating Dynamics under Perpendicularly-Oriented Electric-Field Impulses: A Molecular Dynamics Study

**DOI:** 10.3390/ijms17071133

**Published:** 2016-07-14

**Authors:** Paolo Marracino, Micaela Liberti, Erika Trapani, Christian J. Burnham, Massimiliano Avena, José-Antonio Garate, Francesca Apollonio, Niall J. English

**Affiliations:** 1 Department of Information Engineering, Electronics and Telecommunications, La Sapienza University, 00184 Rome, Italy; erikatrapani@gmail.com (E.T.); massimilianoavena@icloud.com (M.A.); 2 School of Chemical and Bioprocess Engineering, University College Dublin, Belfield, D4 Dublin, Ireland; 3 Computational Biology Laboratory, Life Sciences Foundation, 7750000 Santiago, Chile; garate.j.a@gmail.com; 4 Centro Interdisciplinario de neurociencia de Valparaiso, Universidad de Valparaiso, 05101 Valparaiso, Chile

**Keywords:** water, permeability, molecular dynamics, electric field, aquaporin

## Abstract

Human aquaporin 4 has been studied using molecular dynamics (MD) simulations in the absence and presence of pulses of external static electric fields. The pulses were 10 ns in duration and 0.012–0.065 V/Å in intensity acting along both directions perpendicular to the pores. Water permeability and the dipolar response of all residues of interest (including the selectivity filter) within the pores have been studied. Results showed decreased levels of water osmotic permeability within aquaporin channels during orthogonally-oriented field impulses, although care must be taken with regard to statistical certainty. This can be explained observing enhanced “dipolar flipping” of certain key residues, especially serine 211, histidine 201, arginine 216, histidine 95 and cysteine 178. These residues are placed at the extracellular end of the pore (serine 211, histidine 201, and arginine 216) and at the cytoplasm end (histidine 95 and cysteine 178), with the key role in gating mechanism, hence influencing water permeability.

## 1. Introduction

Aquaporins (AQPs) constitute an extensive family of trans-membrane proteins forming channels that conduct selectively water, as well as other small uncharged molecules (such as glycerol). This selective permeation is a result of osmotic pressure between both sides of the membrane, also serving to exclude very strictly the passage of ions and protons [1,2]. AQPs are in all known lifeforms and are essential for regulating precisely water content in organs and cells. In humans, their defective function is implicated in various pathological conditions, such as nephrogenicdiabetes, insipidus and congenital cataracts [3]. Since their original discovery by Agre et al. [4], several hundred AQPs have been elucidated and characterized [3,5]. In any event, a deeper and more complete understanding of osmotically-driven water permeabilities and fluxes in AQPs is both warranted and essential for progress in medical research to establish more confidently their function and gauge more adeptly their potential involvement in medical conditions. Bearing this goal in mind, water fluxes in AQPs are estimated relatively routinely via reconstitution of channels in liposomes and monitoring changes in volume due to concentrations of an impermeable solute; it may also be possible to estimate diffusive water permeability from isotope-labeling experiments [1,2,4,6,7,8]. To obtain single-channel permeabilities, the AQP density is essential, i.e., the liposome’s precise lipid-to-protein composition. In most cases, this constitutes a significant challenge.

Even knowing the channel densities, obtaining an atomistic-level description of water-transport mechanisms in AQPs is not experimentally feasible, due primarily to the short, nanosecond timescales involved [9,10]. Given these relatively fast kinetics, together with the onset of availability of atomic-resolution AQP structures [11,12,13], molecular dynamics (MD) has become a very valuable tool for gaining theoretical insights into the underlying mechanisms [14,15,16,17,18,19,20,21,22,23,24]. MD studies have considered the characteristics of proton blockage by AQPs [25,26,27,28,29,30], the transport of other solutes [31,32,33,34], the gating of aquaporins [35,36,37] and aquaporin-mediated cell adhesion [38].

In particular, Human Aquaporin 4 (h-AQP4) is abundantly expressed in blood–brain and brain–cerebrospinal fluid interfaces, and is responsible for homeostasis of cerebral water; its function is related to neuropathological disorders such as brain edema, stroke and head injuries [2,3,39]. Recently, the atomic structure of h-AQP4 was resolved by x-ray crystallography at a resolution of 1.8 Å (PDB entry code 3GD8) [40]. As with all AQPs, h-AQP4 forms homo-tetramers in cell membranes, with each functional unit having eight transmembrane helices for which both well-conserved asparagine-proline-alanine (NPA) motifs meet at the pore center.

As integral membrane pore proteins, AQPs are thus considered the heart of the selective regulation conduction of water molecules in and out of the cell; this selectivity mechanism has been long investigated and the conclusion of extensive molecular dynamics/quantum mechanical simulations is that for both the NPA motifs and for the histidine/arginine selectivity filter located on the extracellular side, electrostatics effect dominate water orientation observed in aquaporin channels. [30] Recently, a permeation mechanism has been proposed in which the pore acts as a “two-stage filter”: the first “selectivity filter” (“SF”) (aromatic/arginine region) lies at the extracellular end of the channel, while, at the cytoplasmic end, a second gate composed by histidine 95 and cysteine 178 serves as the other stage [37], wherein a well-defined water dipolar rotation occurs during passage through the channels [24].

Given this, and considering increasing focus on biological effects of electromagnetic fields [41,42,43,44,45,46,47,48,49,50], as well as in nanotechnology and bio-sensing [51,52], there is great interest in investigating effects of external electric fields on molecular/water transport in confined geometries (like nano-pores or aquaporins). In particular, Schoenbach et al. [53] have reported experimental results of pulsed electric field of nanosecond time duration and of the order of MV/m applied to cells. Such pulses allow access to the cell interior through conduction currents flowing through the permeabilized plasma membrane, making possible selective alteration of the cells’ behavior and/or survival.

Garate et al. [54] performed MD simulations of h-AQP4 embedded in a solvated lipid bilayer, and considered the effects of continuously-applied static and alternating electric fields on water transport process, and key features such as single-channel osmotic and diffusive permeabilities. Reale et al. [55] carried out similar simulation of embedded-bilayer h-AQP4, albeit in the absence and presence of nanosecond-scale static and alternating electric-field impulses, together with post-field relaxation, establishing, with the aid of in-field metadynamics, that the dipolar alignment of histidine-201 plays an intimate role in determining gating mechanisms and water flux in external electric fields, especially those oriented parallel with the pore channel. Moreover, in previous work, aside from h-AQP4 [54,55], Garate et al. [56,57,58] observed that water flux through single walled carbon nanotubes (SWCNTs) embedded in solvated lipid membranes is affected by low-intensity static and time-varying electric fields and the effects of dipolar rotation were noted on modulating water flux [54,55,56,57,58].

However, open questions remain from the seminal studies of references [54] and [55] in relation to h-AQP4 behavior in external electric fields. Notably, these include the interplay of the applied fields vis-à-vis the dipolar orientations adopted by all residues, and not just histidine-201 (i.e., the selectivity filter), dipolar alignment in terms of axial field direction. In both references [54] and [55], both axially- and perpendicularly-applied fields were used but with low intensity (0.0065 V/Å), and both manifested important effects. A systematic study of orientational effects on all relevant residues has been considered by Alberga et al. [37], albeit not in the presence of external electric fields; interestingly, they speculate with interest on possible effects thereon by external agents. The application of nanosecond pulses to cells will be expected to give rise to membrane patches, and hence transmembrane h-AQP4 proteins, exposed either to electric fields aligned parallel to the channel and electric fields exposed perpendicular to it.

Enlarging the focus on more residues of the pores will better elucidate the gating mechanism of h-AQP4, both analyzing the selectivity filter (histidine 201 and arginine 216), and also going into deeper details of neighbors like serine 211; even more importantly, this permits a more complete perspective on what has been identified, in absence of external electric field, by Alberga et al. [37] as a second gate in the cytoplasm end, i.e., histidine 95 and cysteine 178.

Bearing in mind especially the subtle matter of field effects on all AQP residues, here we have performed equilibrium and non-equilibrium molecular-dynamics (NEMD) for 10 ns of h-AQP4 in the absence and presence of externally-applied static electric fields applied perpendicularly to the channels. The (peak) intensity of the applied fields has been set at a higher level of 0.012 to 0.065 V/Å to those adopted in previous work [54,55] (0.0065 V/Å). The external-field force on each atom has relatively low magnitude at this intensity vis-à-vis those of inter- and intra-molecular potential’s interactions (around 1%–10%). The relatively short nano-pulse lengths of 10 ns were chosen to agree with lengths typically used in previous work [55], together with experimental trends towards nanosecond field pulses [44]; intense fields are necessary to observe tangible field effects within shorter-duration field pulses. The structural integrity and stability of the membrane itself was not compromised in these in-pulse simulations.

## 2. Results and Discussion

### 2.1. Osmotic Permeability

Following the Einstein approach [59], the self-diffusivity coefficients of the water molecules passing through each channel may be estimated from the center of mass (COM) positions via

(1) $$D=\frac{1}{2}\underset{t\to \infty }{\lim}\frac{\langle {\left[{\vec{r}}_{z}(t_{0}+t)-{\vec{r}}_{z}(t_{0})\right]}^{2}\rangle }{t}$$

from the mean square displacement (MSD) of those water molecules which complete a passage through the channel. Naturally, this limits the maximum duration of the MSD in each of the four pores (denoted A, B, C and D) for which adequate statistics for all such passage events may be gathered. However, it was found from examination of log-log plots of each pores’ water MSD that the self-diffusivities do not develop Fickian behavior, as one might expect; indeed, this phenomenon has been previously studied by Liu et al. [60], Milischuk et al. [61] and Garate and co-workers [54,55] in the context of confined water (with Garate and co-workers studying this in the context of h-AQP4).

**Figure 1:**
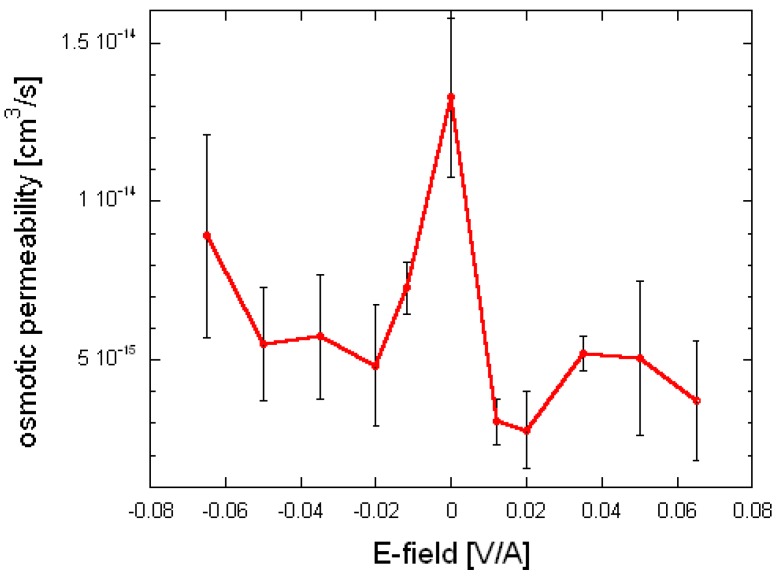
Osmotic permeability for 10 ns static-field pulses along both ±*y*-axis direction as a function of pulse intensity, along with 100 ns-sampled zero-field results.

In order to better describe the behavior of water molecules inside the channels, self-diffusivity values were used for compute the osmotic permeability *p_f_* following the approach of Hashido et al. [21] and Alberga et al. [37]: this is the pre-eminent parameter studied mainly in the literature to characterize AQP water-diffusional behavior in the absence of external electric field. Averaged in-pore osmotic permeability (vis-à-vis the zero-field result) are depicted in Figure 1 as a function of static-field intensity and direction, along with their standard deviation (across the four channels) shown as error bars. The osmotic permeability (*p_f_*) decreases in fields with all intensities applied along both *y*-directions, to the extent that pairwise one-tailed Student’s *t*-tests with respect to the zero-field case indicate increased *p_f_*.

In contrast to references [54] and [55], where the (non-Fickian) self-diffusivities indicated no significant increase, here ANOVA testing [62] confirms that there is indeed a difference between the osmotic permeability ratio. This finding is new with respect to this previous work, probably due to the higher field intensities, that has permitted overcoming the “statistical limit”, or threshold, for tangibly observable “signal-to-noise” ratio. The larger fields employed in the present work (especially in the 0.035–0.065 V/Å range) do not offer scope for excitation due to a ground-state forcefield treatment [63], but certainly allow for greater rotational response of residues’ dipoles to the fields [64,65,66], and consequent rearrangement of hydrogen bonding within the pores (while the membranes remain intact and stable), to decrease the osmotic permeability of water within the pores.

### 2.2. Role of Residues’ Displacement

**Figure 2:**
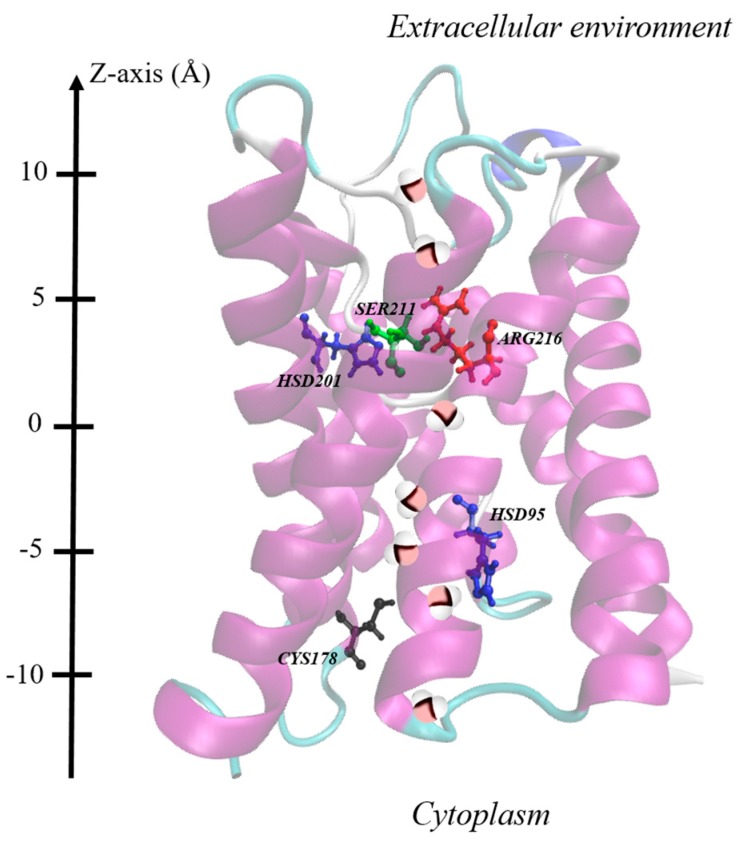
Depiction of layout of the residues considered with the +*z* orientation coinciding with the vertical bottom-to-top direction, for which rearrangements are particularly evident in the regions of the respective pore mouths (see main text). At the upper side (extracellular end), SER-211 (depicted in green), ARG-216 (depicted in red) and HSD-201 (depicted in blue). These latter two residues are usually referred to as the selectivity filter), and, at the lower side (cytoplasm end), CYS-178 (depicted in black) and HSD-95 (depicted in blue). These two have been recently suggested as a gating site by Alberga et al. [37]. The lining of water molecule inside the pore is also represented.

These findings of reduced osmotic permeability raise the natural question of which residues are rearranged in response to the fields: this is defined as a heavy-atom root-mean squared deviation (RMSD) of more than 1 Å with respect to the initial configurations, after 1 ns. In Table 1, we provide just such a systematic overview. An important trend is that residues with larger dipole moments tend to be more amenable to coupling, particularly in more intense fields (as one would expect) [44], regardless of the field direction. Indeed, the broad symmetry of the osmotic-permeability profile with respect to y-axis field direction in Figure 1 is mirrored somewhat in the Table 1 matrix’s more-or-less even-handed distribution of pores in both directions, which undergo substantial rearrangement. Interestingly, and belying the complexity of the AQP system, not all pores undergo appreciable rearrangement in each pore, as presumably pore–pore interactions between the corresponding residues in each pore does not allow all to respond simultaneously. In a key advance from reference [55], which studied in detail on histidine 201 (HSD-201), we see that, serine 211 (SER-211), and arginine 216 (ARG-216), at the extracellular end of the channel, and histidine 95 (HSD-95), and cysteine 178 (CYS-178), at the cytoplasm end of the channel, arguably show an even greater level of field response, with HSD-95, SER-211 and CYS-178 showing a particularly marked response, even stronger than the two residues classically referred to as the selectivity filter (HSD-201 and ARG-216). In Figure 2, we examine a graphic showing the relative positions of these residues, and it becomes readily evident that the residues at the pore “mouths” at the respective ends of the pores appear to respond more readily to the applied fields. This is likely to be attributable to the greater structural rigidity of the centre of the membrane, with the regions in contact with the “bulk” liquid water more free to rotate their dipoles to a great extent vis-à-vis the applied fields. In particular, this is evident in Table 1 for SER-211 at the “upper” gate (constituted also by HSD-201 and ARG-216), and for CYS-178 as the “lower” gate (formed also by HSD-95).

### 2.3. Diverse Dipolar Orientation of Residues

**Figure 3:**
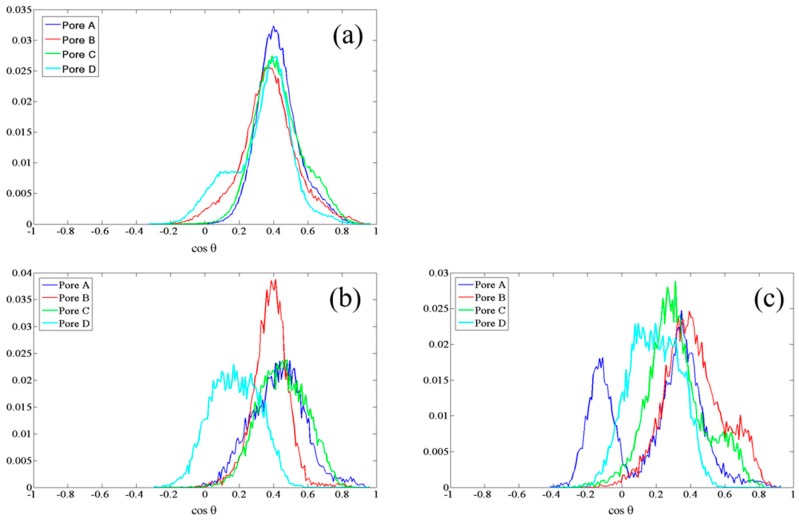

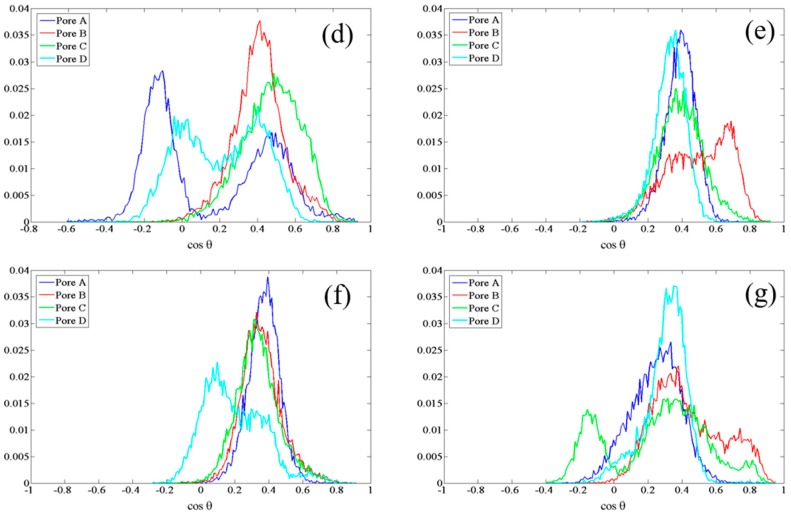
Normalized probability distribution of cosθ in all four pores, for SER-211 in the “upper” gate (see Figure 2) under: (**a**) zero-field; (**b**–**d**) static fields of 0.02, 0.035 and 0.065 V/Å, respectively, along the −*y*-axis; and (**e**–**g**) static fields of 0.02, 0.035 and 0.065 V/Å, respectively, along the +*y*-axis.

**Figure 4:**
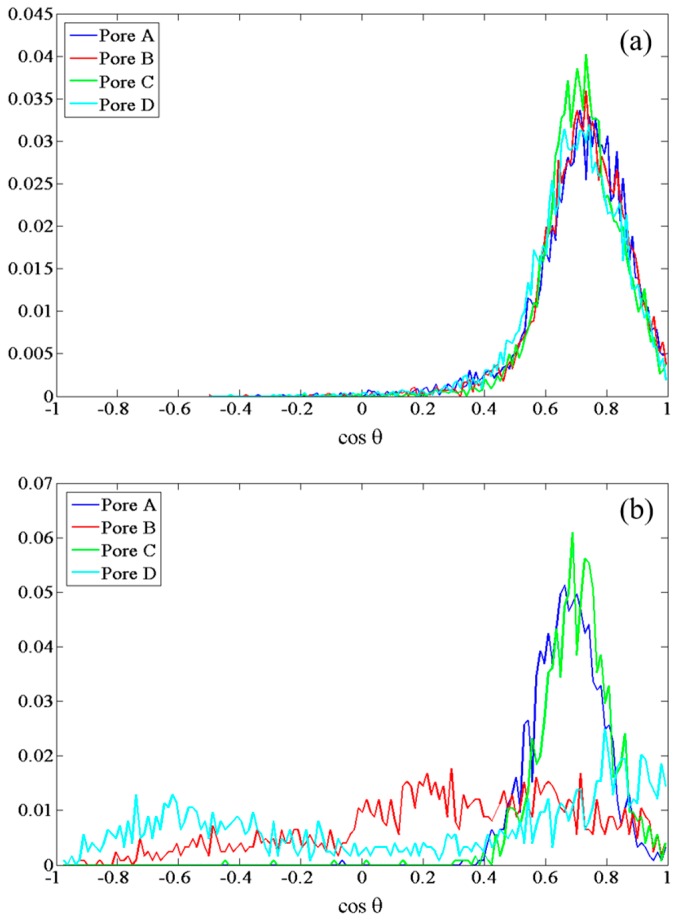
Normalized probability distribution of cosθ, for CYS-178 in the “lower” gate (see Figure 2): (**a**) in all four pores under zero-field conditions; and (**b**) in all four pores in a static field of 0.065 V/Å along the −*y*-axis.

**Figure 5:**
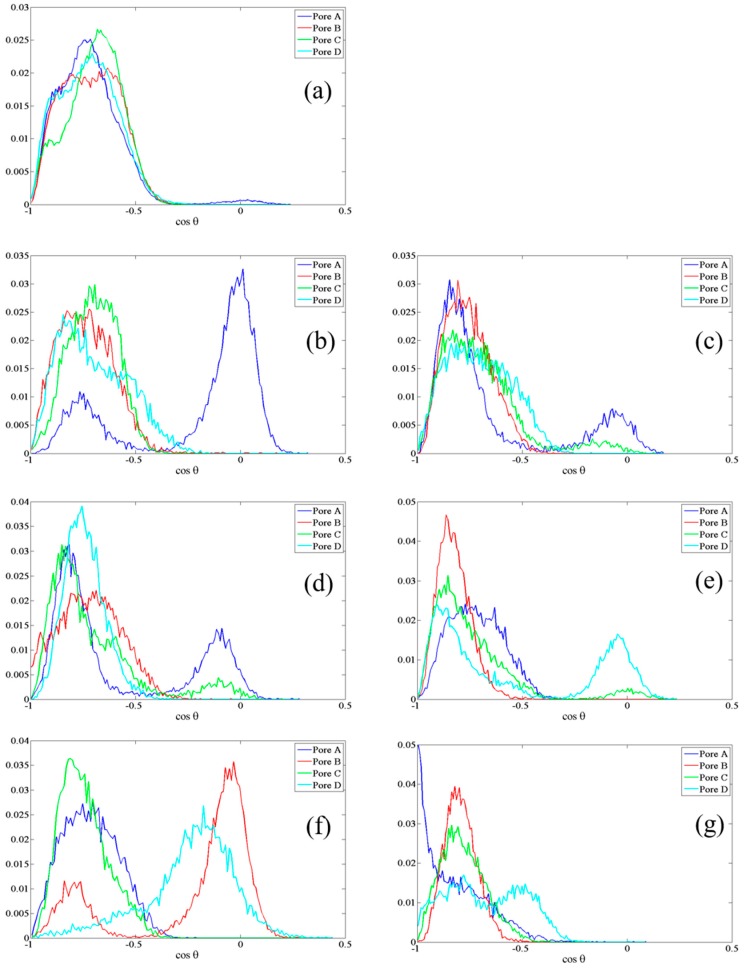
Normalized probability distribution of cosθ in all four pores, in the “lower” gate for HSD-95 (see Figure 2) under: (**a**) zero-field; (**b**–**d**) static fields of 0.02, 0.035 and 0.065 V/Å, respectively, along the −*y*-axis; and (**e**–**g**) static fields of 0.02, 0.035 and 0.065 V/Å, respectively, along the +*y*-axis.

Given this underlying dipolar orientation of residues to induce rearrangements noted above, it behooves a study of field effects directly on the dipolar orientations of these residues, with particular scrutiny afforded to SER-211, HSD-95, and CYS-178. To this end, we examine normalized probability distributions of the (cosine of the) angle (θ) between dipole vectors of these residues with respect to the +*z*-axis—denoted as cosθ. Examining the upper gate first, in the guise of SER-211, Figure 3 shows a systematically larger deviation away from the zero-field distributions as the field intensities increase, as one might expect, with the population of new dipole-orientational states. Interestingly, there is no clear dependence of dipolar-redistribution (in terms of readily-discernible bias to higher or lower cosθ values) with field direction (along either ±*y*-axis). Still, the field effects are rather dramatic, especially at and above 0.035 V/Å. For the lower gate, in the context of CYS-178’s cosθ profile in Figure 4, there is a similar orientational “disruption” evident at and above 0.035 V/Å vis-à-vis the zero-field case (compare Figure 4b with Figure 4a); indeed, this is redolent of the development of bias towards a more “open” CYS-178 state. In this context, the particularly dramatic response of CYS-178 is consistent in Table 1, and in broad agreement with decrease in-pore water osmotic permeability of Figure 1. Given the large response of HSD-95 evident in Table 1, Figure 5 examines the respective cosθ distribution as a function of field direction (±*y*) and intensity, as for SER-211 in Figure 3. In Figure 5, there is somewhat more pronounced dependence on field direction (±*y*) for HSD-95 dipole-orientational-state redistribution with respect to zero-field conditions than for SER-211 (Figure 3): in particular, there is a greater shift to lower cosθ values for fields along the −*y*-direction, although it is difficult to conclude this with statistical certainty (and this proposition does not pass a 90%-threshold *t*-test on comparison of means, for instance). In any event, for all three residues highlighted in Figure 3, Figure 4 and Figure 5, the clear disruption of single orientational states is evident, towards multi-modal distributions, particularly at/above 0.035 V/Å.

### 2.4. Kinetics of Dipole-Orientational Transitions

**Figure 6:**
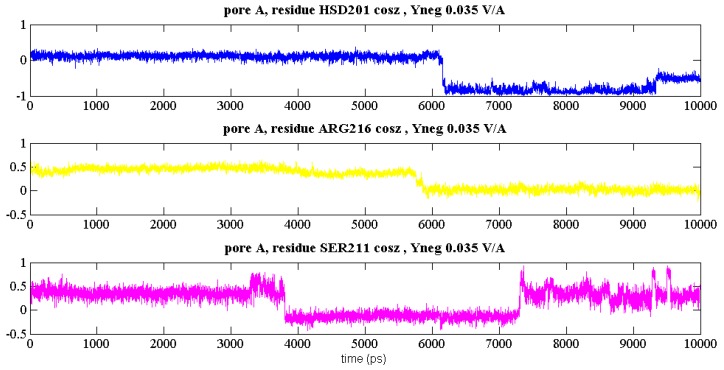
Representative evolution of cosθ in pore A at the “upper” gate for HSD-201, ARG-216 and SER-211 (see Figure 2) under a static field 0.035 V/Å oriented along the −*y*-axis.

It is interesting to explore the kinetics of these dipolar transitions. In Figure 6, representative time evolutions (at the upper gate for pore A) are depicted of HSD-201, ARG-216 and SER-211. The “gateway” status for SER-211 is very clear in Figure 6, with the transition around 3.5 ns serving as a pre-cursor to residues farther along the pore (see Figure 2), with ARG-216 transitioning just before 6 ns and HSD-201 just after this time point. In any event, the rates of transition were found to be typically higher vis-à-vis the zero-field case via single-tailed t-tests, with 90% + significance, once the intensity was at 0.05 V/Å and higher, in either ±*y*-direction. This points to the weakening of hydrogen-bonding arrangements not only serving to distort certain larger-dipole residues, particularly in the mouth areas (see Table 1, Figure 2), and sample new orientational configurations (see Figure 3, Figure 4 and Figure 5), but also points to points to accelerated kinetics of such orientational-state sampling.

## 3. Materials and Methods

### 3.1. Modeling

The computational methodology is largely identical to that of Garate et al. [54] for deterministic (non-equilibrium) MD; however, a brief synopsis will be provided here. The h-AQP4 X-ray crystal structure was obtained from the Protein Data Bank [67] (entry code 3GD8), and the tetramer unit constructed using transformation matrices therein. Missing and hydrogen atoms were added assuming pH 7.5 for protonation states using internal coordinates for CHARMM27 topology [68,69]; histidine protonation states were set at neutral, with the proton positioned on N_δ_. p*K*a calculations were carried out , and no substantial differences between either protonation states (i.e., Nε and N_δ_) were observed. All crystallographic water molecules were retained. The h-AQP4 tetramer was embedded in an equilibrated and solvated palmitoyloleoylphosphatidyl-ethanolamine (POPE) lipid bilayer placed in the *x*-*y* plane; overlapping lipids were removed and a solvation shell of 20 Å was added in the −*z* and *z* directions, while the *z*-axis was set as normal to the bilayer [54]. Na^+^ and Cl^−^ ions were placed randomly in the water to neutralize the system, reaching a final concentration of 55 mM. The final dimensions of the periodic cell were 101 × 101 × 80 Å, consisting of a total of 85,701 atoms [54]. Graphical depictions of the simulated system may be found in references [54] and [55].

### 3.2. Molecular Dynamics

All simulations were performed with the MD program NAMD (developed by the joint collaboration of the Theoretical and Computational Biophysics Group (TCB) and the Parallel Programming Laboratory (PPL) at the University of Illinois at Urbana-Champaign) v2.7 [70,71] with the CHARMM27 potential [68] and the TIP3P water model [72]. The Particle Mesh Ewald [73] method was used for full long-range electrostatics, with a r-RESPA multiple time step decomposition [74] of 1, 2 and 4 fs for bonded, short-range non-bonded, and long-range electrostatic interactions, respectively [54]. All production runs were performed coupled to an NPT reservoir (with constant cross-sectional *x*-*y* surface area) with set points of 1 atm and 298 K using the Nosé–Hoover method [75] and Langevin dynamics for piston fluctuation control [76] with a damping coefficient of 1 ps^−1^. The SHAKE algorithm [77] was applied to constrain bond lengths to all hydrogen atoms. System relaxation is as described in reference [54].

One hundred nanoseconds of production MD was performed under zero-field, equilibrium conditions. Static fields were applied [44] along the −*y* and +*y* directions (with previous studies [54,55] just considering the −*y* case) with NEMD simulations of such orthogonally-applied fields with intensity E_0_ of 0.012, 0.02, 0.035, 0.05 and 0.065 V/Å and time duration of 10 ns. Static fields exert a force over atomic partial charges *ia* defined by $f_{ia}=q_{ia}E_{0}$.

As an aside, Gaussian-type fields with the same peak intensities were also applied along the −*y* and +*y* directions: these had the form of those in reference [78], although no statistically significant differences were found vis-à-vis zero-field simulations—so this shall not be discussed further. For all field conditions, results were averaged over three independent simulations.

Equilibrium MD simulation indicates that local electric field intensities in condensed phases are in the range of around 1.5 to 2.5 V/Å [79,80], giving rise to de facto “signal-to-noise” ratios of around 200:1 to 40:1 for the intrinsic to applied fields in the present work. Field strengths in the 0.1–0.5 V/Å range may be obtained routinely in experiment by applications of potentials of 1 to 5 kV onto tips of radius 10–100 nm [81]. GPU (Graphics Processing Unit) acceleration was applied in NAMD v2.10 [71,82] on an NVIDIA double-precision M2090 platform, and led to a substantial acceleration (approximately 2.5–3-fold) vis-à-vis the equivalent number of CPU-cores (Central Processing Unit cores) on quad-core Intel Xeon nodes connected via sub-microsecond-latency Infiniband. The acceleration was primarily, though not only, in the evaluation of intermediate-range non-bonded van der Waals and electrostatics interactions [58,83].

Although the applied fields will have an important polarization effect at the atomic level, CHARMM27 is a non-polarizable potential, thus the results presented will not account for this effect. Despite this shortcoming, previous works of English et al. [79,80,84] have shown that the effects on liquid water under the influence of e/m-fields are described qualitatively compared to the use of a polarizable potential. Apart from the matter of polarizability per se, Gumbart et al. [85] Mand Casciola et al. [86,87] have shown recently that the application of external electric fields is valid for a proper description of the membrane potential.

### 3.3. Osmotic Permeability

A key quantity that characterizes the transport properties of a water channel is the osmotic permeability *p_f_* measured in cm^3^·s^−1^. This is defined as:

(2) $$j_{s}=p_{f}\Delta C_{s}$$

where *j_s_* is the flux due to a non-permeable solute, and Δ*C_s_* denotes concentration differences. *p_f_* in the absence of any chemical potential difference or an external force is calculated via the collective diffusion model [88]. A collective coordinate, *n_t_*, which encompasses the number all the water molecules within the channel, *S*(*t*) at time *t* is defined in its differential form as

(3) $$d_{n}=\underset{i\in S\left(t\right)}{\sum }dz_{i}/L\left(t\right)$$

where *L*(*t*) is the length of the channel at time t and *dz_i_* is defined as:

(4) $$dz_{i}=\left[z_{i}\left(t\right)-z_{i}\left(t-\delta t\right)\right]$$

with *z_i_* being the *z* coordinate of the *ith* water within *S*(*t*). Integrating *d_n_*:

(5) $$n\left(t\right)=\int_{0}^{t}dt/L\left(t\right)\underset{i\in S\left(t\right)}{\sum }dz_{i}$$

The latter provides for the definition of the diffusion constant *D_n_*, which follows from the mean square displacement (MSD) of *n*(*t*):

(6) $$D_{n}=\langle n^{2}\left(t\right)\rangle /2t$$

measured in *t*^−^. Lastly, *p_f_* is computed by

(7) $$p_{f}=v_{w}D_{n}$$

with *v_w_* the average volume of a water molecule.

## 4. Conclusions

Water osmotic permeability and the dipolar response of all residues within h-AQP4 pores using MD in the absence and presence of pulses of external static electric fields were studied. The pulses were 10 ns in duration and 0.012–0.065 V/Å in intensity, acting along both directions perpendicular to pores. We found enhanced “dipolar flipping” of key residues, especially serine 211, histidine 95 and cysteine 178. The mouths of the pores were more amenable to more pronounced dipolar orientation and distortion, and this led to accelerated sampling of new dipolar states in more intense fields. This work has established the diverse behavior of the various residues lining the aquaporin channels, particularly at their mouths, in terms of influencing water permeability under the influence of external electric fields, and confirms the speculation of Alberga et al. [37] of the heterogeneity of residues’ behavior in the case of some physiochemical perturbation—in this case, an external electric field. In view of the growing importance of electric fields in nanotechnology, medicine and industrial settings, this fundamental mechanistic understanding on such a prototypical transmembrane protein such as h-AQP4 is particularly timely.

## Figures and Tables

**Table 1:** Summary of pores A, B, C, and D, the four aquaporin channels (an example of a single aquaporin channel is reported in Figure 2), which undergo appreciable rearrangement in static-field pulses along the ±*y*-axis. This is defined as a heavy-atom root-mean squared deviation (RMSD) of more than 1 Å with respect to the initial position.

E-Field (V/Å)	−0.065	−0.05	−0.035	−0.02	−0.012	0.012	0.02	0.035	0.05	0.065
ARG-216	A		A	C			B			B, C
ASN-213										
ASN-97	C									
HSD-95	A, C	A	A, C	A		B	C, D	B, D	B	A, D
GLY-94	D							D	D	
GLY-93	D	D					B		C, D	C
HSD-201		A	A						B, D	C
SER-211	A, D	A, D	A, D	D			B	D		A, B, C
PHE-77	A		A	A						
VAL-197										A
ILE-81										
LEU-170				B	A, B					A
ILE-193										A, D
VAL-85								D		A, D
ILE-174										
CYS-178	A, D	A, D	A, D					C	B, C	B, C
